# Optically induced electrothermal microfluidic tweezers in bio-relevant media

**DOI:** 10.1038/s41598-023-35722-3

**Published:** 2023-06-17

**Authors:** Kshitiz Gupta, Hye-Ran Moon, Zhengwei Chen, Bumsoo Han, Nicolas G. Green, Steven T. Wereley

**Affiliations:** 1 School of Mechanical Engineering, Purdue University, West Lafayette, IN USA; 2 School of Electronics and Computer Science, University of Southampton, Southampton, UK

**Keywords:** Fluid dynamics, Microfluidics, Biomedical engineering, Electrical and electronic engineering, Mechanical engineering

## Abstract

Non-contact micro-manipulation tools have enabled invasion-free studies of fragile synthetic particles and biological cells. Rapid electrokinetic patterning (REP) traps target particles/cells, suspended in an electrolyte, on an electrode surface. This entrapment is electrokinetic in nature and thus depends strongly on the suspension medium’s properties. REP has been well characterized for manipulating synthetic particles suspended in low concentration salt solutions (~ 2 mS/m). However, it is not studied as extensively for manipulating biological cells, which introduces an additional level of complexity due to their limited viability in hypotonic media. In this work, we discuss challenges posed by isotonic electrolytes and suggest solutions to enable REP manipulation in bio-relevant media. Various formulations of isotonic media (salt and sugar-based) are tested for their compatibility with REP. REP manipulation is observed in low concentration salt-based media such as 0.1× phosphate buffered saline (PBS) when the device electrodes are passivated with a dielectric layer. We also show manipulation of murine pancreatic cancer cells suspended in a sugar-based (8.5% w/v sucrose and 0.3% w/v dextrose) isotonic medium. The ability to trap mammalian cells and deposit them in custom patterns enables high-impact applications such as determining their biomechanical properties and 3D bioprinting for tissue scaffolding.

## Introduction

Micro-manipulation techniques such as rapid electrokinetic patterning (REP)^1–3^, optical tweezers (OT)^4–6^, opto-electronic tweezers (OET)^7–9^, dielectrophoresis (DEP)^10,11^, hydrodynamic tweezers^12–14^ and many others have gained significant traction in handling micro/nanoparticles and biological cells. Mechanical microgrippers based on pneumatic, electric, magnetic, thermal and many other actuation technologies have been used to physically capture and manipulate microparticles and biological cells^15^. However, these microgrippers are fabricated using complex and expensive photolithography processes. Moreover, the microgrippers apply forces in the order of nanoNewtons or more^16^. In many applications, particles and cells are prone to damage by the physical contact as well as the large manipulation forces of these microgrippers. This vulnerability limits their usage with biological cells. Careful investigation into the mechanical properties of biomolecules can reveal previously unknown bio-mechanical processes. For instance, changes in structure due to sub-picoNewton forces can affect the gene expression in a DNA molecule^17–19^. REP manipulates target particles/cells by trapping them with a microfluidic vortex induced by a uniform AC electric field and localized temperature gradients^20–23^. The electric field is introduced through parallel plate electrodes while the temperature gradient is typically created using a tightly focused laser beam^24,25^ or resistance heaters^26,27^. We have previously demonstrated that REP is capable of creating dynamically tunable stable traps while exerting ultra-small forces (~ femtoNewtons) on the trapped particles^28,29^. The non-invasive nature of electrothermal vortex driven REP traps makes them useful in investigating the behaviors and properties of fragile biological cells. However, biological cells also require specialized medium with closely monitored temperature, humidity, pH, O_2_ and CO_2_ levels to remain viable for long durations of time. In the absence of favorable physiological conditions, cells can undergo cytolysis due to excessive endosmosis or they can shrink due to exosmosis. Most commonly used cell culture and suspension media contain salts (chloride and phosphates of Na and K), amino acids (such as L-glutamine), sugars (sucrose, dextrose, etc.), serums (such as fetal bovine serum) and many other constituents^30,31^. These entities affect the characteristics of the electric double layer formed at the electrode and the cell surfaces, which play a central role in the AC electrokinetic phenomena^32^. The following sections assess how different constituents of cell media affect the REP manipulation mechanism and how those challenges are overcome for handling mammalian cells . Electrochemical properties are essential in understanding the electrokinetic behavior of the medium and the particles/cells. Table 1 summarizes the properties, that are crucial for modelling particle/cell-electrode interaction, of different electrolytes used in this work. Medium c onductivity and zeta potential of 1 µm polystyrene particles in the respective medium are measured using a Malvern Zetasizer NanoZS90. Medium pH is measured using a Thermo Scientific Orion 5 Star meter.

**Table 1:** Electrolyte properties.

Electrolyte	Conductivity (mS/m)	pH	Zeta potential (mV)
0.1 mM KCl	2 ± 0.05	7.10	− 65.1 ± 0.4
Sucrose (8.5%) + Dextrose (0.3%)	1.97 ± 0.01	4.23	− 28.7 ± 0.26
Sucrose (8.5%) + Dextrose (0.3%) + 2 mM GlutaMAX™	15.6 ± 0.2	4.67	− 37 ± 0.83
0.1× PBS	181 ± 10.4	7.19	− 43.67 ± 1.69
1× PBS	1676 ± 52.6	7.45	− 10.5 ± 0.57

### Challenges with AC electrokinetic phenomena in bio-relevant media

**Figure 1:**
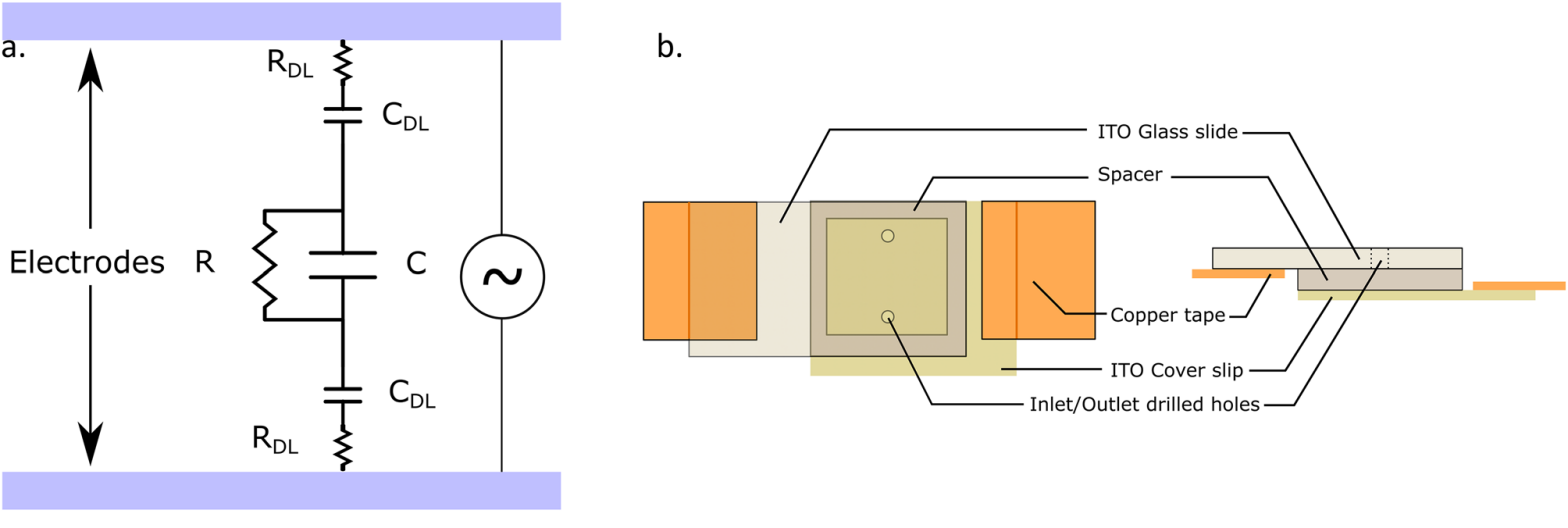
(**a**) RC circuit representation of electric double layer. (**b**) Schematic of a REP chip.

As discussed above, highly specific culture and suspension media that mimic the physiological conditions well are required to keep the biological cells viable. A suspension medium needs to be isotonic to prevent cell lysis due to osmosis. Salts (chloride and phosphates of Na and K) and sugars (sucrose, dextrose, etc.) are the most commonly used constituents for maintaining the osmolarity of cell suspension media. Phosphate buffered saline (PBS) is one such salt-based medium widely used for cell suspensions and has an electrical conductivity of ~ 1.5 S/m (1000× the ~ 2 mS/m aq. KCl solution typically used in REP). The electrolyte, sandwiched between two parallel plate electrodes of a REP chip, can be modelled as a series of capacitors and resistors as shown in Fig. 1a.

However, a high salt concentration (137 mM NaCl, 10 mM Na_2_HPO_4_ and other salts) in PBS can increase the number of reactions (Faradaic and non-Faradaic processes) at the electrode surface. Processes in which charged ions migrate to the electrode surface but there is no charge transfer are called non-Faradaic (capacitive)^33,34^. The ions can be adsorbed or desorbed at the electrode surface but, there is no Faradaic current involved in the process. The adsorbed ions screen the charge of the electric double layer and decrease its thickness. Resultantly, the double layer capacitance is decreased significantly which in turn reduces the relaxation time constant (τ) of the model RC circuit. The equilibrium charge distribution in a smaller capacitor is established quickly and a majority of the applied voltage drops across the double layer. The electric field in the bulk of the electrolyte diminishes significantly which weakens the electrothermal body force driving the REP vortex^32^. Additionally, the increased salt concentration can even collapse the diffuse layer, resulting in non-specific adhesion of the particles with each other and to the electrode surface.

The processes in which charged ions migrate to and from the electrode surface and undergo redox reactions are called Faradaic processes. These reactions are accompanied with Faradaic current due to the movement of ions and exchange of charges at the electrode surface^33,34^. These processes of exchange or adsorption of ions can disrupt the double layer charge distribution, deposit reduced metal ions, generate gas bubbles due to electrolysis and erode electrode material at the reaction sites which limit the stability of a REP trap. Moreover, the electrical conductivity of a salt-based medium under electric field as strong as that used in REP (~ 10^5^ V/m), is significantly higher than the low-salt medium typically used in REP. This can promote concentration of the electric field at the location of electrical contacts with the voltage generator. The electrical potential and the electrical field are not uniformly distributed over the REP chip in such a case. Resultantly, the REP electrothermal vortex and trap can cease to exist. This is mitigated, to some extent, by connecting the copper tape contacts to opposite ends of a REP chip (Fig. 1b). Additionally, the Joule heating effect in low salt concentration media is assumed to be negligible in comparison to the temperature rise due to the optical illumination^35^. However, with high salt concentrations, this source of heat can contribute to the temperature profile significantly. An overall increase in the electrolyte temperature can reduce the temperature gradients created by a tightly focused laser spot and diminish the electrothermal body force driving the REP vortex. The increase in overall temperature is not only detrimental to the REP vortex but can also be harmful to biological cells if it rises above the favorable physiological conditions.

With the above-mentioned limitations, using salt-based cell media can be challenging for micro-manipulation tools based on the electrokinetic phenomena such as REP, DEP and OET. The following sections discuss how a REP chip and the operating conditions can be altered to make such electrokinetic tools viable in bio-relevant media. Passivation of the electrode surface, application of DC offset in the electric field and use of sugar-based medium are explored to enhance manipulation and trapping of biological cells.

### Salt-based media: electrode passivation

High salt concentrations in bio-relevant media, such as PBS, pose many challenges for electrokinetic micro-manipulation tools. The effects of electric field concentration near the location of the copper tape contacts (Fig. 1b) are mitigated by passivating the electrode surface with a dielectric film. A thin film passivation layer acts like a resistor and a small capacitor in series with the double layer and the bulk medium. In one cycle of the AC electric field, as the dielectric film (capacitor) accumulates charge, the electric potential in the conductive electrode material reaches a uniform distribution. It also prevents any Faradaic currents, as charge gets accumulated in the dielectric layer and does not get transferred. This promotes a uniform electric field in the electrolyte even if there is a high salt concentration. A passivation layer should be chemically inert, adhere well to the substrate, and be stable under high electrical fields. Fabrication of dielectric layers often require a baking step and hence it should be able to withstand thermal stresses during heating and cooling processes. A silicon-dioxide (SiO_2_) layer is often grown thermally on a silicon wafer or deposited on other substrates through the processes of chemical vapor deposition (CVD) or physical vapor deposition (PVD). In this work, however, a liquid spin-on-glass (SOG) is used for electrode passivation. An SOG is an oxygen-containing liquid dielectric which is spin-coated on a substrate and baked to form inorganic glass passivation layer. The SOG layer, although, has a high resistivity (~ 10^15^ Ω cm) and creates a significant drop in the bulk electric field. A voltage amplifier is used to compensate for the loss of electric field due to the SOG.

### Sugar-based media: DC offset in the electric field

Many biological applications use sugars, amino acids and serum-based cell media to maintain the osmolarity in contrast to salt-based media^30,31,36,37^. A sucrose (8.5% w/v) and dextrose (0.3% w/v) solution (~ 265 mOsm/L) is made in ultrapure (18.2 MΩ cm) water^38^. This solution has the same osmolarity as 1X PBS. Although, as it is not salt based, a passivated REP chip is not needed for the sugar-based medium. Adding unpolarizable sugar molecules in water reduces its overall permittivity and hence reduces the double layer capacitance. Resultantly, a significant REP vortex is observed only at relatively high applied voltages of 9–10 V_pp_ and 50–80 mW laser powers. As discussed in previous REP articles^39–41^, particle entrapment near the electrode surface increases significantly at low field frequencies (≤ 30 kHz). However, particle entrapment in the sugar-based medium is not observed even at frequencies as low as 10 kHz. A DC offset, introduced in the electric field, is found to enable particle entrapment and manipulation in the sugar-based medium.

## Materials and methods

### REP chip and apparatus

Methylsiloxane 21F (Dielectric constant = 3.9 @1 MHz, Filmtronics) is the spin-on-glass (SOG) used in this work for passivating the ITO coverslips (18 × 18 mm, 8–12 Ω, SPI Supplies) in a REP chip. The coverslip is cleaned by washing in acetone, ethanol, isopropanol, and ultrapure water for 2–3 min. The coverslip is placed in a spin-coater and ~ 50–60 µl SOG is dispensed on it , followed by spinning at 3100 rpm for 15 s. The SOG layer thickness is measured to be 317 ± 19 nm using a Bruker ContourGT-K Interferometer. Subsequently it is baked at 80 °C, 150 °C and 250 °C for 1 min each, to dry the solvent using hotplates. Lastly, it is pyrolyzed in a nitrogen purged furnace (650 series Programmable Muffle Furnace, Cole-Parmer) at 425 °C for 1 h. The temperature in the pyrolysis step was ramped up and down gradually over 10 min to avoid cracks due to thermal stresses. The SOG needs to be stored at room temperature for 24 h before application. It can be filtered using a 100–200 nm Teflon filter if any crystals are found in the container. Readers are referred to the Filmtronics SOG catalog for detailed discussion on the material properties, fabrication process, and troubleshooting. A REP chip is assembled following the procedure presented in supplementary Sect. S1 and as discussed by Mishra, Gupta and Wereley^28^ using the SOG passivated ITO. A Telulex SG-100/A function generator is connected with an amplifier (ADA4870ARR-EBZ, Analog Devices) to compensate for the field strength loss due to the SOG layer. The applied potential is measured using an Agilent DSO1012A oscilloscope. Various suspensions (~ 10^7^ particles/ml) of 1 µm polystyrene beads (FluoroMax microspheres, Thermo Scientific), made in 0.1 mM KCl, 0.1× PBS, 0.25× PBS and 1× PBS, are tested with the REP setup assembled as described above.

The connection sequence of the reference and signal terminals of the function generator, in a purely AC signal, is inconsequential. However, the charge distribution and polarization of the electric double layer is dependent on the terminals’ connection sequence in the presence of a DC offset. In order to make all the data collected consistent and comparable, the bottom (ITO coverslip) electrode of the REP chip is always connected to the reference (negative) terminal of the function generator.

### Mammalian cell culture

KPC2 cells, a murine pancreatic cancer cell line, are developed from pancreatic adenocarcinoma (PDAC) of a genetically engineered mouse model with KRAS and p53 mutations (LSL-KrasG12D/+ , LSL-Trp53R172H/+ , Elastasepr-CreER alleles). The cells were developed and provided by Dr. Stephen Konieczny’s lab at Purdue University^42,43^. The cells are cultured in Roswell Park Memorial Institute (RPMI) 1640 medium supplemented with 5% v/v fetal bovine serum (FBS) and 100 μg/ml penicillin/streptomycin. The cells are harvested by 0.05% trypsin and 0.53 mM EDTA (Life Technologies, CA, USA)^43^ at around 80% confluency. Harvested cells are maintained in an incubator at 37 °C and 5% CO_2_ physiological conditions and suspended in the sugar-based medium (8.5% w/v sucrose and 0.3% w/v dextrose) for REP manipulation.

### Cell viability assay in sugar-based medium

3-(4,5-dimethylthiazol-2-yl)-2,5-diphenyl tetrazolium bromide (MTT) assay (CyQUANT™, Sigma-Aldrich, USA) was used to assess the KPC2 cell viability in the sugar medium. KPC2 cells were initially placed on a 96-well plate (10^4^ cells/well) and cultured for 24 h in the RPMI 1640 medium (supplemented with 5% v/v fetal bovine serum (FBS) and 100 μg/ml penicillin/streptomycin). Then, the cells were washed with PBS three times and subsequently were exposed to the sugar medium for 0, 15 min, 30 min, 1 h, 6 h, and 24 h. As a control group, the cells were exposed to just the culture medium as well for the same time durations. After exposure to the sugar medium, the cells were incubated with 1.2 mM MTT overnight (4–8 h). Afterwards, 0.1 mL isopropanol with 0.04 N HCl was added to each well and mixed thoroughly. Absorbance at 570 nm was measured by a BioTek Epoch plate reader (BioTek, USA). Cell viability is gauged as a percentage of respective control groups.

### Live/dead cell staining assay

To assess the cell viability of KPC2 cells cultured on ITO coated glass substrates with different surface treatments and a control substrate, each substrate is kept in a well on a 6-well plate and 25,000 cells are plated in each well. The cells are allowed to grow for two days, and the viability is assessed by staining nucleic acid using Hoechst 33,342 (Sigma-Aldrich, St. Louis, MO) and nuclei of the dead cells with propidium iodide (Sigma-Aldrich, St. Louis, MO). Nuclear fluorescence is determined with an inverted microscope (Olympus IX71, Japan) using a DAPI, and TRITC filter. Viable cells are quantified as total nuclei counts—dead cell nuclei count and normalized by the viable cells on the control substrate. The error bars in the figures show the standard error of the cell count. The cell viability assay of KPC2 cells is also assessed after being exposed to electric field (10 V_pp_, 100 kHz) by counting live/dead cells by staining live cells with green fluorescence Calcein AM (Life Technologies, CA, USA) and PI.

## Results and discussion

### Salt-based media

**Figure 2:**
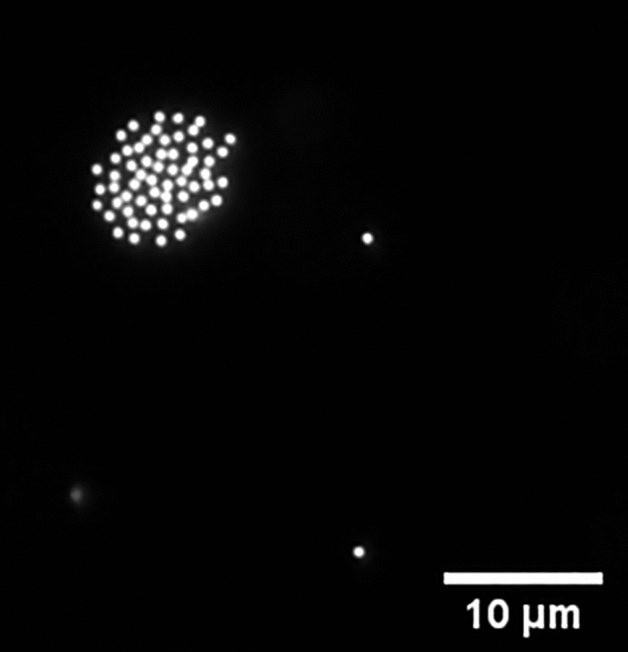
REP trap of 1 μm particles suspended in 0.1 mM aq. KCl solution in a SOG passivated chip.

A suspension of 1 µm polystyrene beads in 0.1 mM KCl solution is used, first, to set a standard of REP trap performance in a passivated chip. An un-passivated chip shows stable particle patterning and manipulation at an applied AC field of 2–10 V_pp_ (~ 10^5^ V_pp_/m), 10–120 kHz. However, due to the high resistivity of the SOG layer, the passivated chip is operated at 20 V_pp_ or above to observe any stable trapping. A REP vortex is observed with the smaller applied voltage (2–10 V_pp_) but a stable trap is observed only with a higher field strength (> 20 V_pp_, 10–50 kHz). Moreover, the SOG layer diminishes the thermal gradient created by the laser and hence the optical power is increased from < 20 mW (typically used for REP) to 80 mW, to form a substantial vortex. Figure 2 shows a circular pattern of 1 µm polystyrene particles suspended in the aq. KCl solution trapped using a passivated chip.

**Figure 3:**
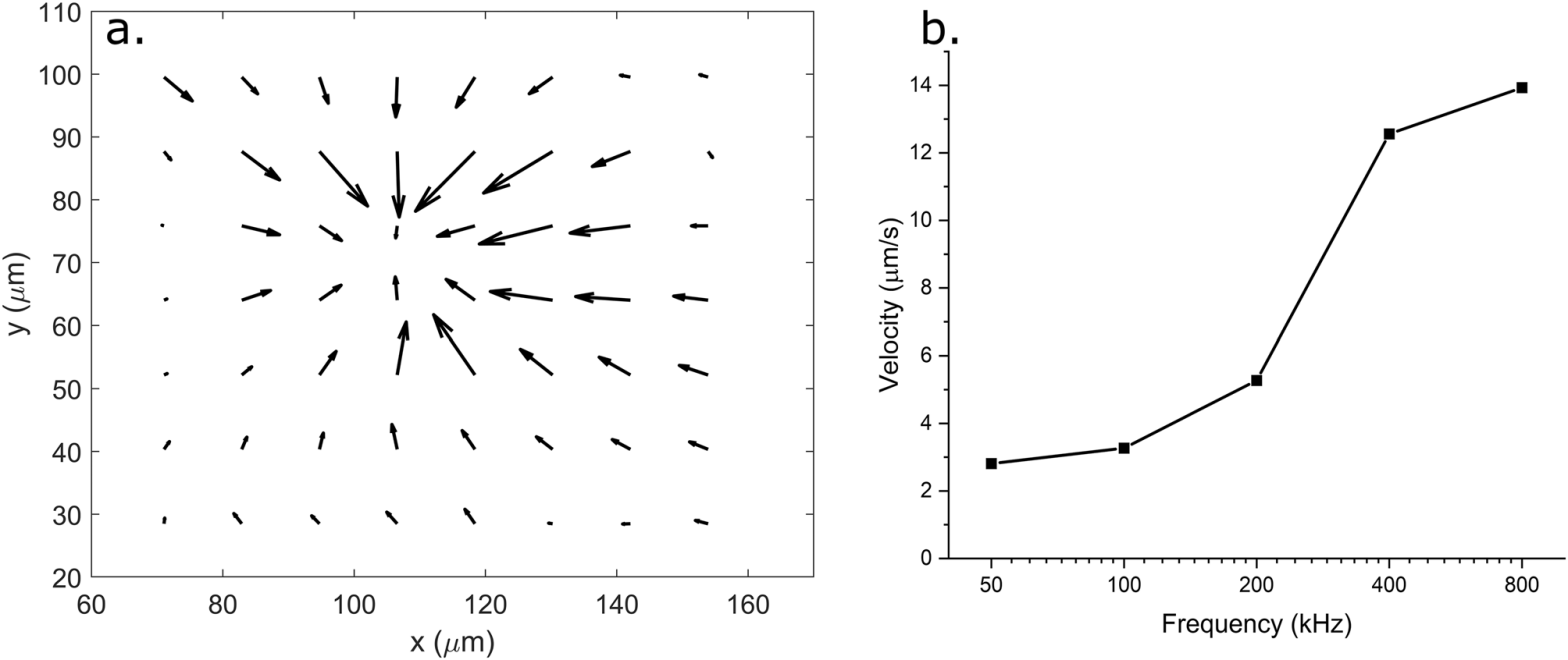
(**a**) Time-averaged velocity field around a REP trap created in a passivated chip with 0.1X PBS in presence of a 24 V_pp_ 100 kHz electric field. The longest arrow corresponds to ~ 3.3 µm/s. (**b**) Maximum velocity of a REP trap in a passivated chip computed by a PIV analysis as a function of field frequency.

Subsequently, the particle suspension in 0.1× PBS is tested in the passivated chip. The voltage applied across the electrodes is increased to 24.4 V_pp_ in comparison to the 20 V_pp_ used with 0.1 mM KCl. This is justified as the increased salt concentration decreases the double layer capacitance and diminishes the electrothermal body force as discussed earlier. As in the case of aq. KCl, particle trapping was observed for frequencies only in the range of 10–50 kHz and just a vortex is observed at higher frequencies. The presence of an additional capacitor (SOG layer) effects the double layer polarization on the electrode surface and hence changes the trapping characteristics. This observation is different from that in un-passivated REP chips which create a stable trap up to ~ 120 kHz, as shown in previous works^20,39,40^. The effect of the field frequency in the vortex formation is studied using a particle image velocimetry (PIV) analysis. The frequency is varied from 50 to 800 kHz and a time-averaged velocity field is computed^44–46^. Figures 3a,b show the time-averaged velocity field for the 100 kHz case and the effect of electric field frequency on the velocity field (as a representative of vortex strength), respectively.

Figure 3a shows that the velocity field in a REP vortex created by a laser spot is circular in shape and radially inwards. It is also observed, as expected, that the velocity magnitude is small at the trap center, increases in the radially outwards direction and decreases again far away from the trap center. The maximum velocity measured in the vortex is plotted in Fig. 3b to represent REP vortex strength. The vortex strength initially increases exponentially with frequency and then approaches saturation. This is consistent with the typical behavior of bulk potential in an electrolyte with a dielectric layer, as the operating conditions reach the charge relaxation frequency of the dielectric. From Fig. 3b above, it is observed that the charge relaxation frequency for the SOG layer is ~ 1 MHz. The electrokinetic behavior of the vortex, given by Eq. (1) below, becomes majorly dielectric above the charge relaxation frequency as the second term on the right-hand-side becomes dominant^35^. The Coulombic effects (term 1 on the right-hand-side decrease with increasing frequency and the vortex eventually changes the direction of its circulation. In Eq. 1, $\langle {f}_{ET}\rangle$ is the time-averaged electrothermal body force driving the vortex, ${\varepsilon }_{m}$ and ${\sigma }_{m}$ are permittivity and electrical conductivity of the medium, $\alpha ={\varepsilon }_{m}^{-1}\left(\frac{\partial {\varepsilon }_{m}}{\partial T}\right)$, $\beta ={\sigma }_{m}^{-1}\left(\frac{\partial {\sigma }_{m}}{\partial T}\right)$, $T$ is the absolute temperature, $E$ is the electric field and ${E}^{*}$ is its complex conjugate, and $\omega$ is the angular electric field frequency.

1 $$\langle f_{ET} \rangle = \frac{{\varepsilon_{m} }}{2}\left[ {\left( {\alpha - \beta } \right)\frac{\nabla T \cdot E}{{1 + \left( {\omega \left( {\varepsilon_{m} /\sigma_{m} } \right)} \right)^{2} }}E^{*} - \frac{1}{2}\alpha \left| E \right|^{2} \nabla T} \right]$$

The suspensions in 0.25× and 1× PBS are also tested in the passivated chip. However, the electrothermal vortex is not observed even at increased applied voltages (30 V_pp_), higher laser powers (~ 200 mW) and a wide range of frequencies (10 kHz–10 MHz). In frequencies < 1 MHz, most of the voltage is dropped across the SOG layer. The slightest of surface defects such as a pinhole or chipped off ITO layer buried under the passivation layer can concentrate the electric field and induce electrolysis. Applying a voltage more than 30 V_pp_ resulted in excessive bubble formation and local electroosmotic vortices near defects which further disrupted the REP vortex. Additionally, high laser powers burned holes in the electrode surface further exacerbating the bubble and vortex formation. Lastly, the high salt concentration decreases the thickness of the double layer on the particles as well. The electric double layers play a crucial role in the stability of colloidal suspensions. Reduced double layer thickness, due to charge screening, promotes permanent adhesion of the particles to each other and to the electrode surface. Hence, it is concluded that high salt concentration media are detrimental to REP trapping and manipulation.

### Mammalian cell micro-manipulation in sugar-based media

**Figure 4:**
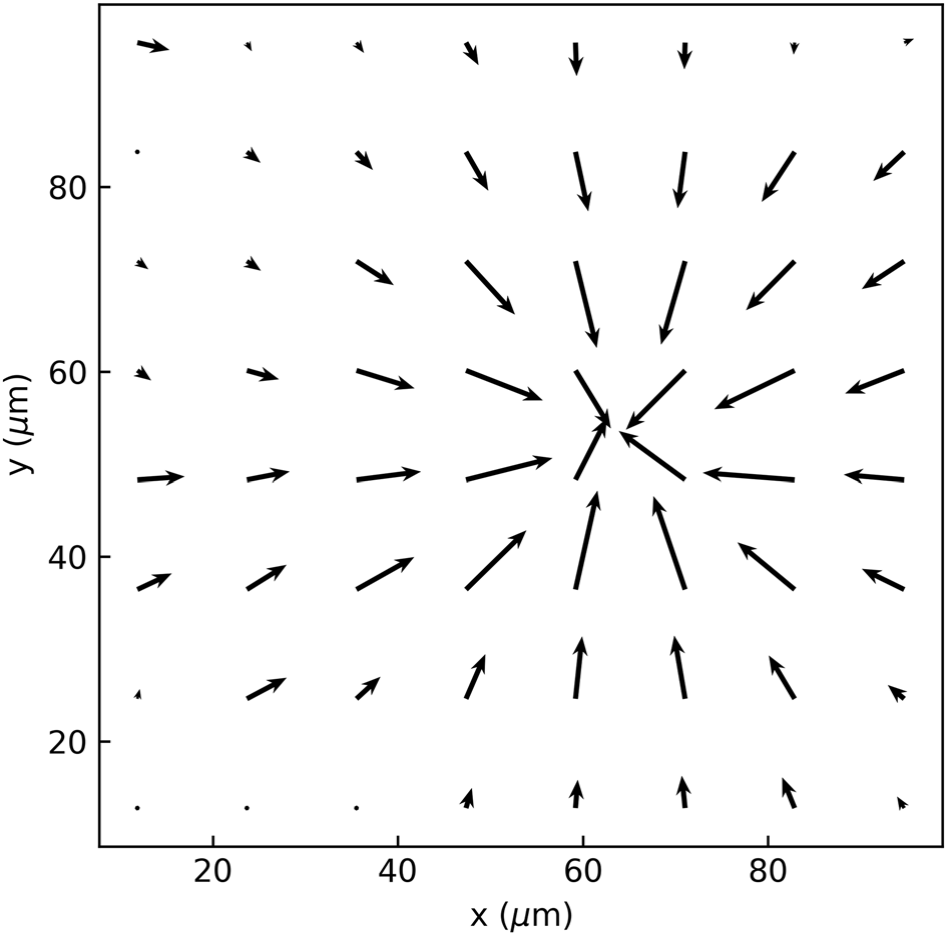
Time-averaged velocity field around a REP trap created with sugar-based isotonic medium in presence of a 10 V_pp_–30 kHz electric field and an 80 mW laser spot. The longest arrow corresponds to ~ 14 µm/s.

The observations from previous section enhance the understanding of REP patterning and manipulation in bio-relevant media. The electrokinetic mechanisms cease to operate in high salt concentration media while most of the biological cells are not viable in hypotonic media. Hence, a sugar-based isotonic medium is used to ensure cell viability as well as that the REP vortex is not hindered significantly. Figure 4 shows the axisymmetric radially-inward velocity field of a REP vortex created in the sugar-based isotonic medium computed using a time-averaged PIV analysis. The vortex was created using a 10 V_pp_–30 kHz electric field and an 80 mW laser spot. It is observed that the maximum velocity, along the plane of the electrodes, is ~ 14 µm/s which is larger than that observed in the salt-based medium around similar frequencies. This observation is explained by the loss in the net electric field strength and temperature gradients in the latter case due to the passivation layer (SOG). Only a vortex is observed in the sugar-based medium and particles are not trapped. Although, introducing a DC offset in the electric field is found to enable REP trapping in the sugar medium. However, studying the effect of the DC component on the nature of a REP trap is beyond the scope of this work. This work focusses on using REP for mammalian cell manipulation and a future article will discuss the effects of DC offset on REP traps.

**Figure 5:**
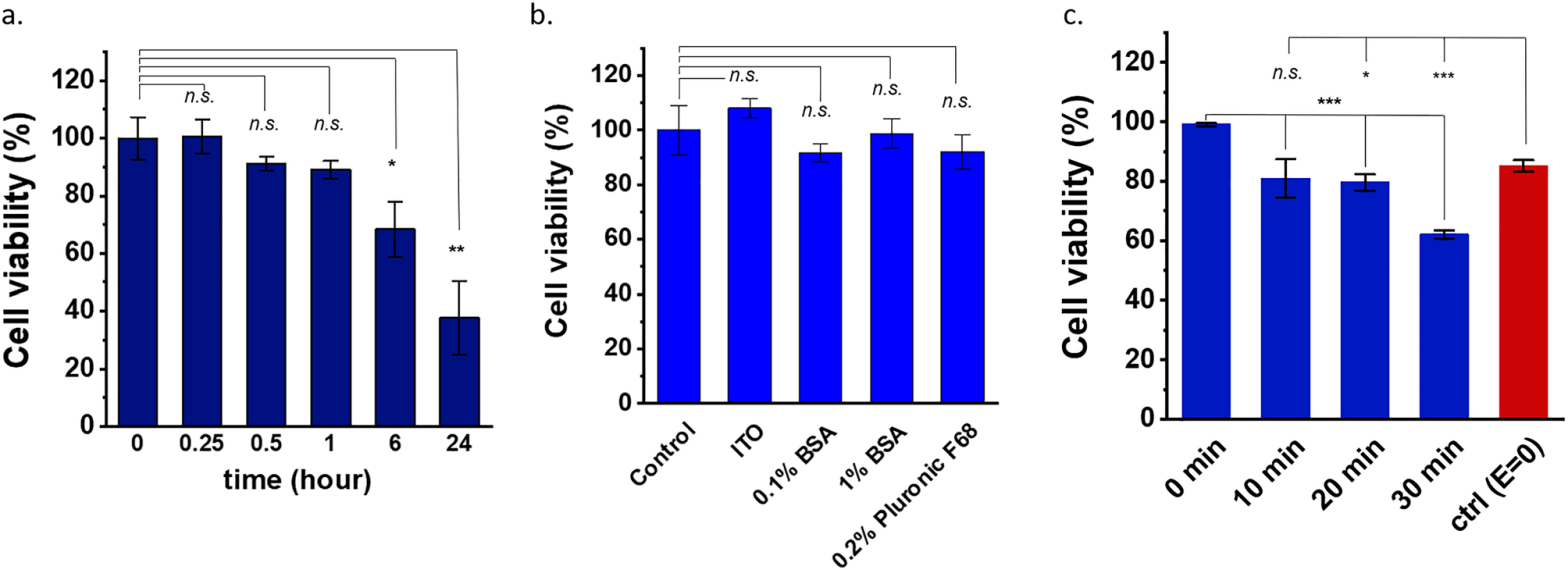
(**a**) Viability of KPC2 cells suspended in the sugar medium for different durations, presented as a percentage of the control group (0 h). (**b**) Live/dead cell viability test on ITO substrates with different surface treatments after two days in regular culture conditions. Cell counts were normalized by the control group. Bars: mean ± std. error (n = 3). (**c**) Cell viability after exposure to an electric field (10 V_pp_, 100 kHz) presented as a percentage of the 0 min case. The control (red bar with E = 0) is a repetition of the 0.5 h case in panel a. Bars: mean ± standard deviation (n ≥ 4). *p < .05, **p < .01, ***p < .001, and *n.s.* indicates no statistically significant difference with respect to the control showing *p* > .05 (Student t-test).

The viability of the cells in the sugar medium is assessed by MTT assay. KPC2 cell growth gradually reduces in the sugar medium but they are relatively viable (~ 90%) up to an hour, as shown in Fig. 5a. Additionally, the viability of the cells is assessed on ITO coated glass substrates with different surface treatments and a control substrate (glass coverslip) using a live/dead cell staining assay. ITO coverslips treated with 0.1% bovine serum albumin (BSA), 1% BSA, 0.2% pluronic F68 solutions and an uncoated ITO coverslip are tested for cell viability in addition to the control. The said solutions are coated on clean ITO coverslips, which are left to dry overnight. The treated substrates are gently washed with ultrapure water to remove excess solution. The surface treatments are performed to prevent non-specific cell adhesion to the REP electrodes during manipulation experiments. The normalized cell count in Fig. 5b shows that the cells are equally viable on all the substrates and surface treatments. Consequently, the cell viability is not affected by any surface coating procedure showing no statistical significance compared to control glass substrate. The viability of cells after being exposed to the electric field (10 V_pp_, 100 kHz) is assessed as well and is presented in Fig. 5c. Even though particle trapping without a DC offset is not seen, a steady REP vortex is observed in the sugar-based media for a wide range of field frequencies. Hence, a 100 kHz field is used for the viability test to minimize non-specific cell adhesion on the electrodes. It is found that the cell viability under electric field decreases with time however, it is > 60% for up to 30 min of exposure. REP entrapment, manipulation and deposition tasks are carried out in a time period of 0.5–1 h. Another control (red bar in Fig. 5c) is assessed in which the cells are exposed to the sugar media in the REP chip for 30 min but without any electric field. This is essentially a repeat of the case presented in Fig. 5a and it is found that the viability is consistent with the said case. It should be noted that the standard deviation in Fig. 5c is calculated by measuring cell count in 4–10 different regions in a single REP chip. Many applications use GlutaMAX™ to improve cell health and viability. However, the additional NaCl from the supplement results in increased non-specific cell/particle adhesion to the electrodes. Hence, it is concluded REP is a viable option for KPC2 cell manipulation as long as the sugar medium is replaced with the culture medium as soon as they are patterned and deposited minimizing electric field exposure to the cells during the process.

**Figure 6:**
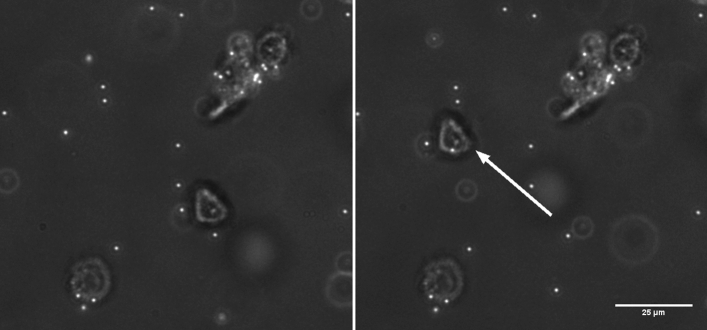
Manipulation of a KPC2 cell using REP. The panels are image frames taken from the supplementary Video SV1. The white arrow shows the translation of a single cell under the influence of a REP trap. 1 μm particles (white dots) are used as tracers for the REP vortex. Scale bar = 25 µm is common for both the panels.

KPC2 cells are separated from the culture medium by centrifugation at 125 g for 5 min and then suspended in the sugar-based medium at a concentration of 2 × 10^6^ cells/ml. The solution is supplied in a REP chip fabricated using uncoated ITO substrates. The chip is subjected to an electric field of 20 V_pp_ (− 200 mV DC offset), 20 kHz and a trap is created using a 40 mW laser spot. Figure 6 and supplementary Video SV1 show the manipulation of a single cell trapped using REP. A single cell is trapped and moved around permanently stuck cells and polystyrene tracer particles. Freely moving tracer particles, in Video SV1, are being translated towards the cell before being swept back into the bulk medium. This shows that the cell is indeed trapped at the center of the REP trap. Note that Video SV1 is sped up 4-times. Stable trapping and manipulation is observed using a laser spot of lesser power (40 mW) than what is used to trap polystyrene particles (50–80 mW) suspended in the same medium as the cells. It is observed that the cells are swept away from the electrode surface, by the REP vortex, into the bulk medium at higher laser powers. This is explained by the larger drag force experienced by the cells due to their size in comparison to polystyrene particles. Moreover, unlike polystyrene particles, cells are not deposited permanently on the electrode surface at low field frequency or high values of DC offset (~ 2 V). Although, some non-specific adhesion was observed on the electrode surface. Additionally, as discussed in our earlier work^28^, REP entrapment in the electrode plane is purely hydrodynamic. A KPC2 cell, much larger than the polystyrene particles, is approximately the same size as the REP trap. While translating, it is more likely to lose the cell as it may fall out of the REP trap’s region of influence. Resultantly, the cells are manipulated at a much slower speed than 1 µm particles. In video SV1 and Fig. 6, the cell is translated over a distance of 45 µm at a speed of ~ 1.3 µm/s. The manipulation speed can be enhanced by increasing the laser spot size which in turn increases the trap size and creating steeper temperature gradients which increase hydrodynamic drag-based trapping force. These observations suggest that the cell-electrode interaction is weak and they are primarily trapped in the potential well created by the drag force of the REP vortex and gravity. It is speculated that the double layer polarization of the cells is not as strong as the polystyrene particles due to their larger size (~ 20 µm). Moreover, unlike a dielectric particle such as a polystyrene bead, a biological cell is much more complex in its structure and its response to an electric field. A biological cell is made of multiple layers of different materials, each of which has a different response to an electric field. Additional comments on modelling the cell-electrode interaction can be found in the supplementary Sect. S2. Further investigation is suggested to enhance patterning and 3D bioprinting capabilities of REP.

## Conclusion

This work extends the non-invasive micromanipulation capabilities of rapid electrokinetic pattering (REP) to bio-relevant media and mammalian cells. It is observed that salt-based isotonic suspension media, such as PBS, can be detrimental to the REP vortex strength. Moreover, they can cause non-specific adhesion globally on the electrode surface. Some effects of the high salt concentration are mitigated by passivating the electrode surface with a thin dielectric layer. Although, passivation enables REP manipulation in low concentration salt-based media (~ 0.1× PBS), it requires the electrical field strength and the laser power to be drastically increased to do so. Alternatively, a sugar-based (8.5% w/v sucrose and 0.3% w/v dextrose) isotonic medium is used to avoid the complexity posed by salts. It is found that KPC2 murine pancreatic cancer cells are viable in the sugar medium for the time scales typical in REP manipulation (0.5–1 h). Additionally, they are compatible with ITO electrodes and various surface treatments used to prevent non-specific permanent adhesion for at least 2 days. As a result of the preceding investigations, single cancer-cells are successfully manipulated with REP for the first time. The speed of cell manipulation with REP was somewhat slower than what is typically used for polystyrene particles. Additionally, the cells did not get permanently deposited on the electrode surface near the trap center at low electric field frequencies (< 10 kHz) unlike synthetic particles. This is attributed to the dielectric behavior of the shell-like structure of cells which is different from the solid particles typically used in REP manipulation.

## Supplementary Information

Supplementary Information 1.

Supplementary Video 1.

## Data Availability

The data generated and analyzed in this study is available from the corresponding author on reasonable request.
