# Supplementary material for: Optically induced electrothermal microfluidic tweezers in bio-relevant media
Source: Sci Rep. 2023 Jun 17;13:9819. doi: 10.1038/s41598-023-35722-3 (PMC10276874; doi:10.1038/s41598-023-35722-3)
Supplement: Supplementary file 1 — Supplementary Information 1. [file 41598_2023_35722_MOESM1_ESM.docx]

Optically induced electro-thermal microfluidic tweezers in bio-relevant media

Supplementary Information

Kshitiz Gupta^1^, Hye-ran Moon^1^, Zhengwei Chen^1^, Bumsoo Han^1^, Nicolas G. Green^2^, and Steven T. Wereley^1*^

^1^School of Mechanical Engineering, Purdue University, West Lafayette, IN, USA

^2^School of Electronics and Computer Science, University of Southampton, Southampton, UK

**S1. Passivated REP chip fabrication**

Passivating ITO coverslip using spin-on-glass (SOG):

1. Clean the ITO coverslips (18x18 mm, 8-12 Ω, SPI Supplies) by washing in acetone, ethanol, isopropanol, and ultrapure water for 2 – 3 minutes each and dry it with pressurized nitrogen.
2. Place in a spin-coater (SCS 6808P Spinner) and dispense 50 – 60 µl Methylsiloxane 21F (Dielectric constant = 3.9 @1 MHz, Filmtronics) SOG on it.
3. Ramp-up the spin-coater to 500 rpm in 5 sec followed by a ramp-up to 3100 rpm in 5 sec.
4. Dwell at 3100 rpm for 15 sec and ramp-down to 0 rpm in a reverse fashion as the ramp-up procedure. Note that a sacrificial layer of a photoresist or a piece of 1 mil tape should be used on a small region of the coverslip to lift off the SOG after spin coating. This provides access to the conductive ITO for electrical connections.
5. Prepare 3 hotplates at 80°C, 150°C and 250°C and bake the coated coverslip for 1 minute each to dry the solvent. Remove the piece of sacrificial tape to expose the conductive ITO.
6. Pyrolyze the coated coverslip in a nitrogen purged furnace (650 series Programmable Muffle Furnace, Cole-Parmer) at 425°C for 1 hour. Ramp-up and ramp-down the temperature gradually over 10 minutes to avoid cracks due to thermal stresses.

REP chip assembly:

1. Cut a 25x25 mm ITO coated glass slide (25x75 mm, 8-12 Ω, SPI Supplies) using a diamond scribe pencil (SPI Supplies).
2. Drill two holes ~5-7 mm away from edge for inlet and outlet ports in the cut ITO glass slide using a 1 mm diamond glass cutting drill bit with a Dremel 3000 drill. Note that all the processing should be done on the glass side to avoid damage to the ITO layer.
3. Wash and dry the cut and drilled ITO glass slide using the same process as the coverslip.
4. Stick the ITO coverslip and the drilled glass slide together with a rectangular spacer made using a 2 mil double sided tape (8171CL optically clear adhesive, 3M). Ensure that the drilled holes are inside the microfluidic cavity. Note that the glass slide should not fully cover the coverslip and some conductive region should be easily accessible for making electrical connections.
5. Make electrical connections at the conductive (ITO) sides of both the electrodes using single-sided copper foil tape (3M) for connecting with the function generator (Telulex SG-100/A).

**S2. Shell-like model for mammalian cell-electrode interaction**

KPC2 cells show significantly different REP trapping characteristics in comparison to polystyrene particles. This can be explained by their size and complex internal microstructure which are different from that of polystyrene particles. This changes their response to Stokes’ drag due to the REP vortex and their interaction with the electrodes under oscillatory electric fields.

Mammalian cells typically have a size of ~10 - 15 µm. As the Stokes' drag increases linearly with the diameter of a particle (or cell), a REP vortex exerts a drag force which is 10 times larger on a KPC2 cell than that on a 1 µm particle. The particle (or cell)-electrode interaction under oscillating electric field depends on the extent of its polarization. The polarization of the electric double layer (EDL) is governed by the Clausius-Mossotti factor$K_{CM}$ :

| $K_{CM}=\frac{\epsilon_{p}^{*}- \epsilon_{m}^{*}}{\epsilon_{p}^{*}+2\epsilon_{m}^{*}}$. | ( 1 ) |
| --- | --- |

Here, $\epsilon_{p}^{*}$ and $\epsilon_{m}^{*}$ are complex permittivity of the particle and the suspending medium respectively. For a solid homogeneous polystyrene particle, the electrical conductivity is calculated as:

| $\epsilon^{*}= \epsilon_{0}\epsilon-j\frac{\sigma}{\omega} ; (j= \sqrt{-1})$ | ( 2 ) |
| --- | --- |

where, $\sigma$ is the electrical conductivity and $\omega=2\pi f$ is the angular frequency of the electric field.

The mammalian cells, however, have a more complex and flexible microstructure [1,2]. The common approach is to use a multi-shell model to represent its morphological structure. However, we use a single-shell model (cell nucleus and membrane) to calculate the simplified effective complex permittivity:

| $\epsilon_{eff}^{*}=\epsilon_{s}^{*}\frac{\left( \frac{R_{0}}{R_{i}} \right)^{3}+2\left( \frac{\epsilon_{r,p}^{*}-\epsilon_{r,s}^{*}}{\epsilon_{r,p}^{*}+2\epsilon_{r,s}^{*}} \right)}{\left( \frac{R_{0}}{R_{i}} \right)^{3}-\left( \frac{\epsilon_{r,p}^{*}-\epsilon_{r,s}^{*}}{\epsilon_{r,p}^{*}+2\epsilon_{r,s}^{*}} \right)}$ | ( 3 ) |
| --- | --- |

Here, $R_{0}$and $R_{i}$ are the outer and inner radius of the single-shell cell model, respectively and $\epsilon_{r,p}^{*},$ $\epsilon_{r,s}^{*}$ are the complex permittivity of the nucleus and cell membrane respectively.


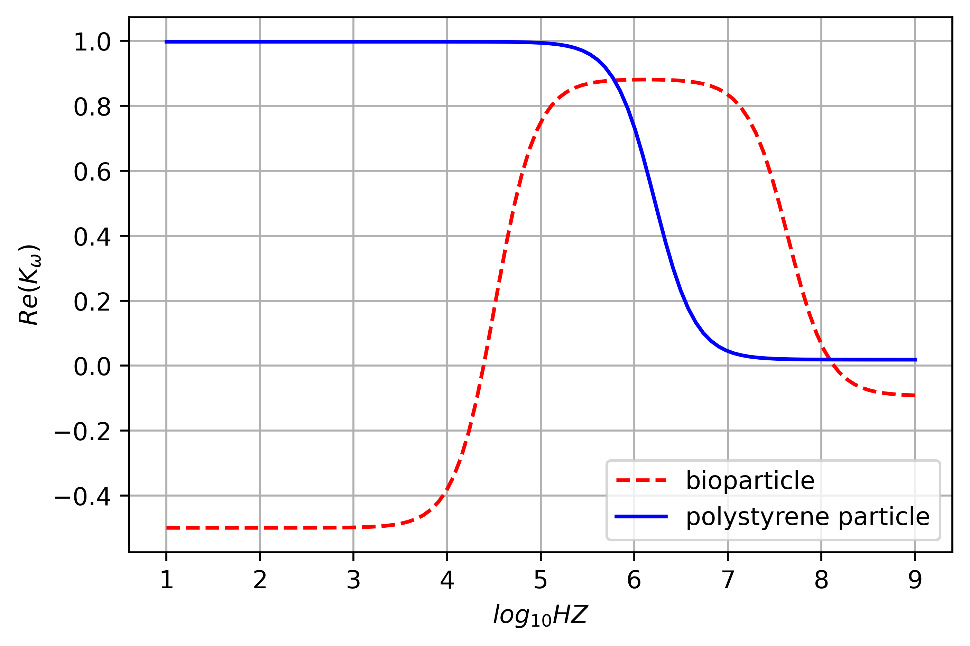


Figure S1. ${Re[K}_{CM}]$ vs frequency for a polystyrene particle and a single shell model cell in sucrose (8.5% w/v) – dextrose (0.3% w/v) solution

Table S1. Parameters used to compute $K_{CM}$[3].

| **Parameter** | **Value** | **Units** |
| --- | --- | --- |
| Electrical conductivity of medium | $1.97$ | $mS/m$ |
| Relative permittivity of medium | $78$ | - |
| Diameter of polystyrene particle | $1$ | $\mu m$ |
| Electrical conductivity of polystyrene particle | $1\times{10}^{-3}$ | $mS/m$ |
| Relative permittivity of polystyrene particle | $2.5$ | - |
| Thickness of cell membrane | 0.02 | $\mu m$ |
| Electrical conductivity of cell membrane | $1\times{10}^{-5}$ | $mS/m$ |
| Relative permittivity of cell membrane | $10$ | - |
| Thickness of nucleus | 15 | $\mu m$ |
| Electrical conductivity of nucleus | 0.5 | $S/m$ |
| Relative permittivity of cell nucleus | 60 | - |

The cell-electrode interaction can be tuned by controlling $K_{CM}$, by selecting the frequency of the electric field. For polystyrene beads in sucrose (8.5% w/v) – dextrose (0.3% w/v) solution, $Re\left[ K_{CM} \right]$ is always positive while the bioparticle modeled with a single-shell has two relaxation frequencies as shown in Fig. S1. Parameters used to calculate $Re\left[ K_{CM} \right]$ are provided in Table S1. The electrical frequency that is typically used for creating a REP vortex is in the range of $10-100 kHz$. The magnitude of $Re\left[ K_{CM} \right]$ for bioparticles, in this range of frequencies, is smaller than that for polystyrene particles which leads to a weaker cell-electrode interaction. Hence, a weaker REP vortex is needed to achieve a stable trap for cells in comparison to particles.

**SV1. Supplementary Video: Manipulation of a single KPC2 cell using REP.**

The supplementary video shows micromanipulation of a single KPC2 cell suspended in a sugar-based isotonic media. The cell is trapped using a rapid electrokinetic patterning (REP) vortex and is translated along the ITO electrode of the REP chip. 1 µm polystyrene particles (white dots) trace the fluid flow and show the cell is at the center of the trap. The trapped cell is being moved around other cells and particles permanently stuck on the ITO.

**References**

1. Zhao, Y., Hu, S. & Wang, Q. Study on the assembly and separation of biological cell by optically induced dielectrophoretic technology. *Microfluid Nanofluidics 2013 17:2* **17**, 287–294 (2013).

2. Chen, Q. & Yuan, Y. J. A review of polystyrene bead manipulation by dielectrophoresis. *RSC Adv.* **9**, 4963–4981 (2019).

3. Morgan, H. & Green, N. *AC Electrokinetics: Colloids and Nanoparticles*. (Research Studies Press Limited, 2003).
